# Preparing surgeons for the modern operating theatre: insights from a national survey on technology use and readiness

**DOI:** 10.3389/fsurg.2025.1686653

**Published:** 2025-11-14

**Authors:** Adam F. Roche, Eoghan Burke, Dara O. Kavanagh, Darach Crimmins, Christina A. Fleming, Niall J. McInerney, Dara O’Keeffe, Pablo Javier Villanueva, Gulam Zilani, Vincent Healy, Donncha O’Brien, Clare McCloskey, Daragh Moneley, Claire M. Condron

**Affiliations:** 1 RCSI SIM Centre for Simulation Education and Research, RCSI University of Medicine and Health Sciences, Dublin, Ireland; 2 Department of Surgical Affairs, RCSI University of Medicine and Health Sciences, Dublin, Ireland; 3 Department of Neurosurgery, Temple Street Hospital, Dublin, Ireland; 4 Department of Colorectal Surgery, Limerick University Hospital and School of Medicine University of Limerick, Limerick, Ireland; 5 Department of Otolaryngology, Mater Misericordiae University Hospital, Dublin, Ireland; 6 Department of Neurosurgery, Hospital Regional de San Martin de los Andes, Neuquen, Argentina; 7 Department of Neurosurgery, Beaumont Hospital, Dublin, Ireland; 8 Department of Ophthalmology, Sligo General Hospital, Sligo, Ireland; 9 Department of Vascular Surgery, Beaumont Hospital, Dublin, Ireland

**Keywords:** surgical education, simulation, curriculum develeopment, equipment training, patient safety, operative safety, neurosurgery, multidisciplinary simulation

## Abstract

**Introduction:**

Rapid advances in surgical technology require formal training in the use of devices and equipment, yet curricula rarely address technology, equipment, and consumables (TEC) proficiency in a systematic way. This study evaluated current TEC training practices, perceived needs, and barriers among consultant-level surgical educators in Ireland and used these findings to develop a national TEC Toolkit framework rooted in contemporary educational theory.

**Method:**

A national cross-specialty survey was distributed to consultant surgical educators, gathering quantitative ratings and qualitative insights regarding TEC education, barriers to readiness, and preferred educational strategies. The twenty-item survey was mapped to Kern's curriculum development framework and constructivist principles. Responses were analysed descriptively and thematically, and Kruskal–Wallis tests compared ratings across three specialty groupings.

**Results:**

Thirty-three of 39 educators responded (85%). Fifty-six percent reported having witnessed patient safety risks or workflow issues due to trainee unfamiliarity with TEC. A strong majority (over 90%) endorsed simulation-based training, hands-on workshops, and competency assessment as essential components of TEC education. Barriers included limited protected time, inconsistent access to TEC, and lack of curricular integration. Qualitative themes highlighted the need for practical skills development, multidisciplinary simulation, digital resources, and ongoing programme refinement. These priorities informed the proposed TEC Toolkit, which integrates simulation, multimedia modules, and structured assessment.

**Conclusion:**

Surgical educators across Ireland overwhelmingly support structured, simulation-driven TEC training as an essential element of modern surgical safety. The proposed TEC Toolkit model offers a practical, evidence-based blueprint to improve TEC literacy and patient safety. Implementing and evaluating this toolkit will help to address current gaps and prepare trainees for the complexities of today's technology-rich operating theatres.

## Introduction

**Figure 1:**
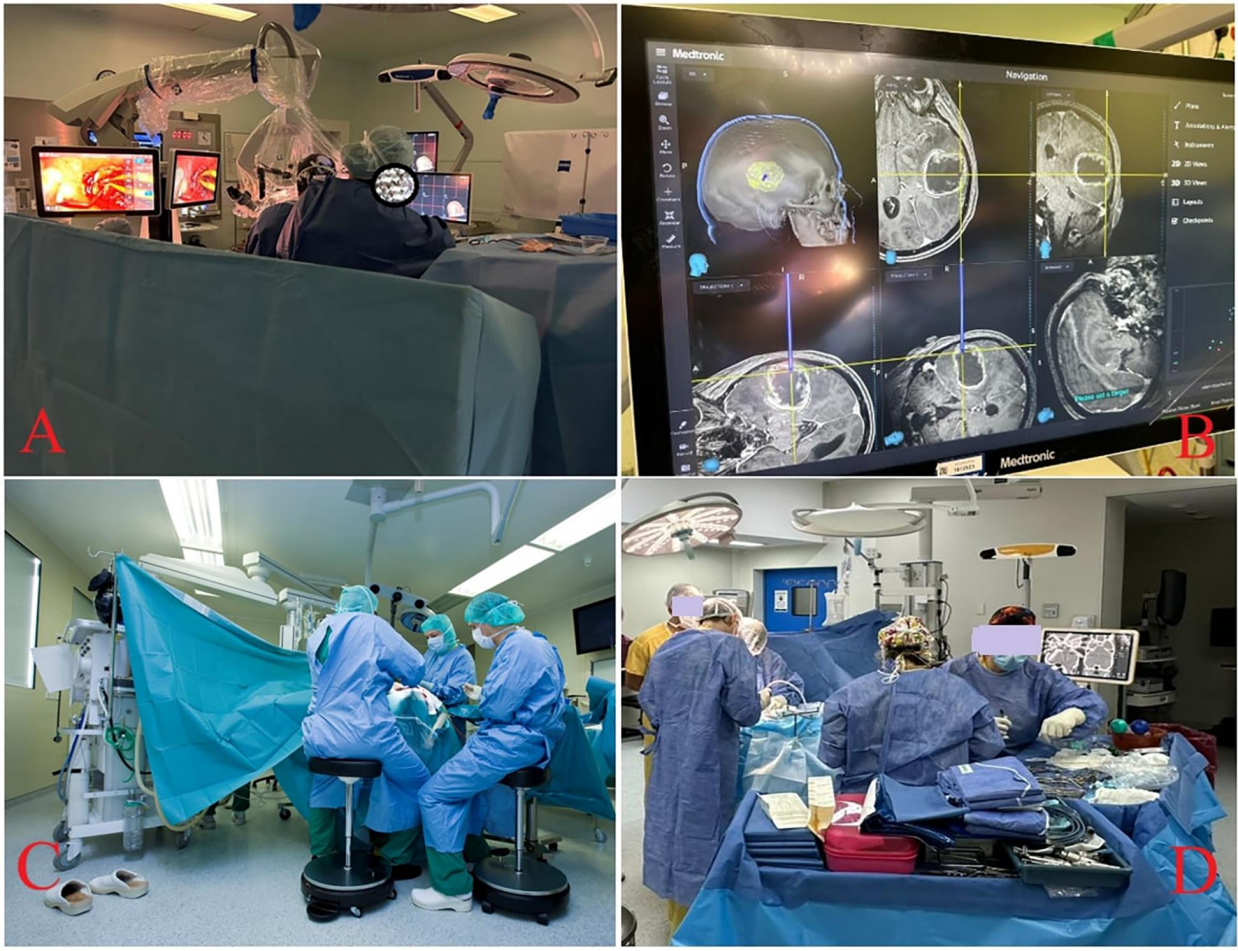
Technological integration in neurosurgery training. **(A,B)** Advanced image-guided neurosurgical setup. Panel **(A)** shows the integration of surgical microscopes, intraoperative displays, and navigation systems, while Panel **(B)** offers a close-up of the neuronavigation interface used for trajectory planning and real-time localisation during cranial procedures. **(C)** Adjustable stools, optimal posture, and microscope positioning during prolonged neurosurgical procedures. **(D)** The array of TEC that require procedural familiarity.

Surgical education has traditionally focused on developing both technical and non-technical skills through conventional training pathways, complemented by structured simulation-based training (SBT) (1). Although technology, equipment and consumable (TEC) operation and familiarity logically fall within the domain of technical skills, they have arguably not been prioritised to the extent required in formal surgical education. The practical challenges of using surgical TEC are often underemphasised, despite being essential to technical competence. As the range and complexity of TEC continue to expand, this oversight has contributed to a critical gap: many training programmes fail to explicitly address TEC proficiency and technological readiness (2), leaving trainees to acquire these important skills informally and under pressure in the operating theatre (OT), as alluded to in Figure 1.

Insufficient TEC familiarity places both patients and trainees at risk,contributing to user anxiety and stress (3), impaired performance, and preventable errors (4). Despite growing evidence that SBT accelerates competency with procedural TEC (5), surgical training curricula don't always address routine TEC handling, selection and troubleshooting. For instance, although operative microscopes are critical for precision in specialties such as maxillofacial surgery, ophthalmology, otolaryngology (ENT) and neurosurgery, their practical application is not always incorporated as a dedicated component of formal surgical training. Consequently, trainees don't always obtain structured guidance on nuanced critical skills such as ergonomic setup, magnification selection, focusing, and hand-eye coordination, and sometimes develop proficiency informally.

A review of malpractice claims found that technical incompetence accounted for 58% of trainee-involved errors (6), though TEC misuse is often obscured within this broad classification due to limitations in reporting systems. Despite their prevalence, TEC-related errors are inconsistently reported. Institutional mechanisms for capturing such incidents are often underutilised and lack standardisation, making it difficult to quantify a truer extent of TEC-related performance gaps in surgical training (7). These findings highlight the need for improved reporting structures to inform targeted training and enhance operative safety.

To address the growing gap in surgical training on TEC readiness, we conducted a national consensus-based study of senior surgical educators in Ireland to evaluate current practices, identify curricular limitations, and gather expert recommendations. Findings informed the development of the “TEC Toolkit”—a curriculum-integrated, simulation-driven recommendations combining multidisciplinary SBT, hands-on workshops, and TEC-specific learning resources. Designed to replace *ad hoc* exposure with structured, deliberate training (8), the toolkit aims to build practical TEC literacy and situational fluency, with iterative updates guided by trainee feedback, technological advances, and outcomes-based data.

Although this study was conducted in the Republic of Ireland (RoI), the educational challenges related to TEC proficiency are widely recognised across international surgical training contexts (9). The survey findings reported here informed the development of the TEC Toolkit, which is presented alongside recommendations for its proposed integration into surgical training curricula.

## Methods

### Study design and participants

This study used a mixed methods design, incorporating both quantitative and qualitative components. A national survey was conducted among surgical educators across 12 surgical specialties in the RoI to evaluate current practices, perceived needs, and support for TEC training. All participants were recruited through purposive sampling (10) and were consultant-level senior surgical educators, currently or formerly serving in roles such as Training Programme Director (TPD) or SBT lead, with direct responsibility for postgraduate training and skills development. For the majority of specialties, invitation and survey administration were coordinated through the administration department of the Department of Surgical Affairs (DoSA) at Royal College of Surgeons Ireland (RCSI), which acts as gatekeeper for these training programmes. Obstetrics and gynaecology (OBGYN), as well as ophthalmic surgery, were included in this survey due to their regular use of the OT and reliance on complex surgical technologies, despite not being among the 10 core surgical specialties formally recognised by the Intercollegiate Surgical Curriculum Programme (ISCP) (11). OBGYN educators were recruited in coordination with the Royal College of Physicians of Ireland (RCPI), which governs postgraduate specialist training in these disciplines. Ophthalmic surgical educators were recruited in coordination with the Irish College of Ophthalmology (ICO). This approach ensured comprehensive access to relevant consultant educators across both colleges and all major surgical specialties. This study was granted ethical approval by the Ethics Committee of RCSI University of Medicine and Health Sciences (review code 202507013). Participation was voluntary and implied consent was provided by completion and submission of the anonymous online survey.

### Survey development

The 20-question survey was developed inductively, informed by constructivist learning theory (12), which values experiential and reflective practitioner perspectives, and guided by Kern's six-step model for curriculum development (13) a systematic framework for identifying educational needs, setting objectives, and aligning instructional strategies. These two frameworks complement one another: constructivism provides the theoretical foundation for how learners acquire and apply knowledge, while Kern's model offers a structured process to translate those learning principles into curriculum design and assessment strategies. Together, they ensured the survey was both pedagogically grounded and practically relevant.

To ensure both clinical relevance and educational appropriateness, two co-authors contributed to the design and review of survey items: one a consultant neurosurgeon and experienced educator (PJV), the other a senior general surgical trainee recently completing specialist training (EB). Their complementary insights ensured the questionnaire reflected both training experience and teaching priorities. The survey was then piloted for clarity and relevance by two additional authors (AR, CC), and each item was explicitly mapped to the relevant stage of Kern's framework and associated learning principles.

### Quantitative analysis

Respondents rated all closed-ended items using a 5-point Likert scale (1 = strongly disagree to 5 = strongly agree). Descriptive statistics were used to calculate the median and interquartile range (IQR). A minimum response rate of 80% was targeted, aligning with recognised benchmarks for rigorous survey research and supporting the potential for population-level generalisation (14). At the end of the survey, open-text fields invited participants to provide free-text comments on perceived gaps, training challenges, and recommendations, which were qualitatively reviewed to identify common themes.

To evaluate potential differences between surgical specialties, respondents were grouped into three clusters reflecting practice focus: (1) Precision & Microsurgical (neurosurgery, ophthalmology, oral & maxillofacial surgery, ENT), (2) General & Endoscopic Visceral (general, vascular, urology, OBGYN, paediatric surgery), and (3) Reconstructive & Musculoskeletal (trauma & orthopaedics, plastic surgery, cardiothoracic surgery). Kruskal–Wallis tests compared responses across these groups for seven preselected survey items addressing key aspects of TEC training, resources, and multidisciplinary practice; other survey questions were not included in this subgroup analysis. All statistical analyses were performed using the Stata Now 19.5 software package (StataCorp, College Station, TX, USA).

### Qualitative analysis

In addition to quantitative items, the survey included optional open-text fields where participants could share reflections, training challenges, and recommendations. These free-text responses were analysed using an inductive thematic analysis approach. Two authors (AR, CC) independently reviewed and open-coded the responses to identify initial patterns. Codes were compared and refined through discussion, and discrepancies were resolved by consensus. Codes were then grouped into higher-level themes that reflected recurring priorities across the dataset.

To enhance transparency, a codebook was developed to document the coding process, including code definitions, example quotations, and corresponding themes (Supplementary File S1). This structured record supports reproducibility and aligns with established qualitative research standards. The final analysis yielded three overarching themes, each illustrated with representative quotations selected for clarity and relevance. This dual-coding approach and use of a codebook enhanced analytical rigour and helped reduce individual bias in interpretation.

## Results

Of the 39 participants invited, 33 completed the survey, yielding an 85% response rate. Participant demographics are shown in Table 1, and survey responses are summarised in Supplementary File S2. Overall, respondents strongly endorsed the need for structured TEC training, with a median Likert score of 4 (IQR 4–5) across most items. Agreement levels ranged from 68%–91% for statements supporting SBT, while less than 10% expressed neutrality or disagreement. Median responses were consistent across specialty groups.

**Table 1:** Participant demographics.

Gender Distribution (M: F)	23: 10
Age (Median & IQR)	51 (46, 57)
Survey response rate per surgical speciality	Cardiothoracic (6%), ENT (6%), General Surgery (6%), Neurosurgery (12%), OBGYN (16%), Ophthalmic Surgery (6%), Oral Maxillofacial (6%), Paediatric Surgery (9%), Plastic & Reconstructive Surgery (6%), Trauma & Orthopaedics (12%), Urology (6%), Vascular (9%)
Years as surgical educator/mentor (Median & IQR)	15 (10, 20)

In addition to quantitative data, participants provided open-ended responses that offered deeper insights into TEC-related challenges and training needs. Thematic analysis of these free-text comments revealed three overarching themes: (1) the impact of TEC inexperience on surgical safety and workflow, (2) widespread support for structured, simulation-based TEC training, and (3) institutional and curricular gaps limiting effective TEC education. Notably, 56% of respondents described instances in which lack of familiarity with commonly used equipment compromised patient safety or disrupted operative flow. These included near-misses, procedural delays, and elevated intraoperative stress. Representative quotations are presented below to illustrate these themes and enrich interpretation of the survey findings.

### Theme 1: impact of TEC inexperience on surgical safety and workflow

Participants described incidents where trainees' unfamiliarity with surgical technology delayed procedures or created potential safety risks.

One participant described, “*A patient required a particular device, but the trainee had never used it before despite their level of training, so I was called in to provide urgent, real-time teaching*.” Another recalled, “*Unfamiliarity with the setup of basic equipment delayed the case and created stress within the team.*” A third participant reflected, “*The inability to efficiently carry out relatively simple tasks using technology can invariably delay the progress of the surgical procedure*.” Another participant recalled, “*a trainee attempted to insert a resectoscope without first using the obturator”*, posing a risk of patient harm.

### Theme 2: strong support for structured simulation-based TEC training

Participants broadly support integrated, hands-on TEC training that mirrors real theatre environments, delivered through deliberate, protected simulation.

One participant remarked, “*Defined, structured, regular, stepwise simulation is essential given the contemporary approaches to surgical training*.” Another noted, “*A compulsory simulation-based component for the surgical curriculum and certificate of completion of training (CCT) requirements is long overdue*.” Others highlighted practical formats for delivery, suggesting “*video-based modules on device setup, usage, and common pitfalls*,” and “*dry-run simulations with nursing staff to build familiarity outside the operating theatre*.”.

### Theme 3: institutional and curricular barriers to effective TEC education

Participants frequently identified systemic obstacles that limit the consistent delivery of TEC training. Many pointed to time pressures, resource limitations, and insufficient curricular integration as key barriers.

One participant commented, “*Time on the back table with nursing staff and exposure to dry-run simulations would help, but there isn't enough protected time for this*.” Another noted, “*There's limited access to up-to-date equipment, which hampers our ability to teach relevant skills*.” Concerns around standardisation were also expressed: “*Simulation training varies significantly across units, there's no structured approach*.” These reflections underscore the need for national guidance, institutional support, and protected training time to enable more consistent and effective TEC education.

### Comparison of surgical specialty groups

Responses were additionally analysed by grouping specialties into three clusters reflecting practice focus: Precision & Microsurgical, General & Endoscopic Visceral, and Reconstructive & Musculoskeletal. For each of the seven selected survey items, there were no statistically significant differences in median ratings between the groups (all *p* > 0.05 by Kruskal–Wallis test). Supplementary File S3 presents the median (IQR) for each group and survey item. This suggests that attitudes, perceived barriers, and supports for TEC training are broadly consistent across different surgical disciplines.

## Discussion

This national survey of consultant-level surgical educators underscores a persistent gap in structured training for TEC within surgical education. Although the study focused on the Irish context, the issues identified, including increasing TEC complexity, limited curricular integration, and patient safety risks, are consistently reflected in international literature. Across health systems, curricula emphasise technical and non-technical skills, yet TEC proficiency remains informal, inconsistently taught, and seldom evaluated. This observation is consistent with international initiatives that highlight the need for structured, simulation-based, and TEC-focused training. For example, the American College of Surgeons (ACS) has developed the Accredited Education Institutes (AEI) Programme to incorporate simulation, faculty development, and competency assessment within surgical education (15). Similarly, the UK Joint Committee on Surgical Training (JCST) and its Specialty Advisory Committees promote simulation integration within the ISCP (16). These international developments reflect a shared movement toward competency-based TEC education, supporting the broader relevance of the findings presented in this study.

Both quantitative and qualitative findings revealed strong support for mandatory, simulation-based TEC training. Educators described how TEC unfamiliarity leads to near-misses, operative delays, and team stress, echoing international concerns that training is lagging behind the pace of technological change. Key barriers identified included limited protected time, uneven access to up-to-date TEC, and inadequate curricular integration. Selecting the correct TEC component in an operative case can sometimes be challenging, especially when an array of options are available and uncertainty arises due to time pressures, fear of making mistakes, and the desire to impress senior colleagues (17). Addressing barriers such as time constraints and inconsistent TEC access requires coordinated institutional and policy-level reform. At the institutional level, allocating protected time for SBT that incorporates TEC teaching within consultant job plans or structured SBT schedules can help offset competing clinical demands. Equitable access to up-to-date surgical TEC across training sites could be facilitated through centralised procurement or regionally managed simulation resources. Collaboration with industry to deliver standardised TEC familiarisation programmes can further reduce variation in trainee exposure. At the policy level, accrediting bodies and training colleges could reinforce accountability by formally recognising TEC competence as a defined curricular outcome, linked to existing training standards and funding requirements (18). Embedding these expectations within national training frameworks would legitimise TEC readiness as an educational priority and promote sustainability through system-level accountability. Meaningful reform in surgical education depends on multi-layered institutional commitment that extends beyond individual programmes to strengthen the structural and cultural foundations of training (19).

In neurosurgery, shunt systems present a high degree of technical variation: valves may be fixed or adjustable via external magnets, with or without anti-siphon mechanisms; ventricular catheters may terminate in the frontal or parietal horns; and distal ends may drain into the peritoneal cavity, pleural space, or venous system. Some configurations use a “Y” connection to drain multiple sites, while others bypass the ventricles entirely to manage cysts. Without structured, repeated training on these variations outside the OT, trainees may perhaps progress to more autonomous stages of training without comprehensively developing all aspects of the fluency required to confidently select, assemble, and troubleshoot these devices (20). Such gaps in preparation can compromise the educational value of such operative opportunities.

Some trainees may be able to set up operating microscopes efficiently, while others require extended assistance, which may prompt senior colleagues to intervene and unintentionally limit learning within the presumed organic, holistic environment of the OT. Basic tasks such as adjusting the chair to the correct height or aligning the eyepieces can become sources of stress when attempted for the first time under the pressures of the OT. Deliberate, structured practice in low-pressure environments is essential for reducing cognitive load (8), particularly the extraneous load caused by unfamiliarity with TEC setup or procedural sequencing. In vascular surgery, for example, uncertainty around stent selection and configuration may persist until late in training, highlighting the cognitive demands placed on learners and reinforcing the need for targeted TEC education across all surgical disciplines. Similarly, in gastrointestinal surgery, trainees often hesitate when using circular bowel staplers, especially in developing an intuitive sense of the force required for safe and effective use. By enabling repeated, feedback-informed rehearsal, simulation aligns with the principles of deliberate practice—allowing learners to refine technique, build fluency, and internalise performance standards outside the time-pressured OT.

Improving TEC training does not require a complete overhaul of existing SBT practices. Instead, adopting a whole-task approach (21), where trainees engage in the full procedural workflow including TEC selection, setup, troubleshooting, and post-operative steps, can enhance both TEC fluency and contextual understanding. SBT that replicates real clinical sequences—such as patient preparation, anaesthetic administration, and equipment configuration—helps embed technical skills within the cognitive and technological demands of modern surgery (22). This approach supports the development of transferable skills (23) and reduces the risk of TEC-related errors during live procedures. Structured exposure to TEC challenges in a low-pressure, controlled environment allows trainees to better anticipate and manage problems as they arise. Transitioning from isolated task training to full-context procedural simulation also aligns with constructivist learning theory (13) and better prepares surgeons for independent practice in technology-rich operative settings. Consistent with prior research linking TEC-handling competence to fewer adverse events and improved workflow efficiency, our findings support the inclusion of TEC-specific simulation within patient safety curricula (24).The proposed TEC Toolkit, comprising hands-on SBT, multidisciplinary workshops, and digital resources, offers a targeted and theory-informed solution. Rooted in constructivist learning theory and guided by Kern's six-step curriculum development framework, the toolkit positions TEC literacy as a core, assessable competency. By providing regular, protected opportunities for simulated TEC practice, it aims to reduce cognitive burden (24) in the modern OT, enhance operative confidence, and improve safety.

**Figure 2:**
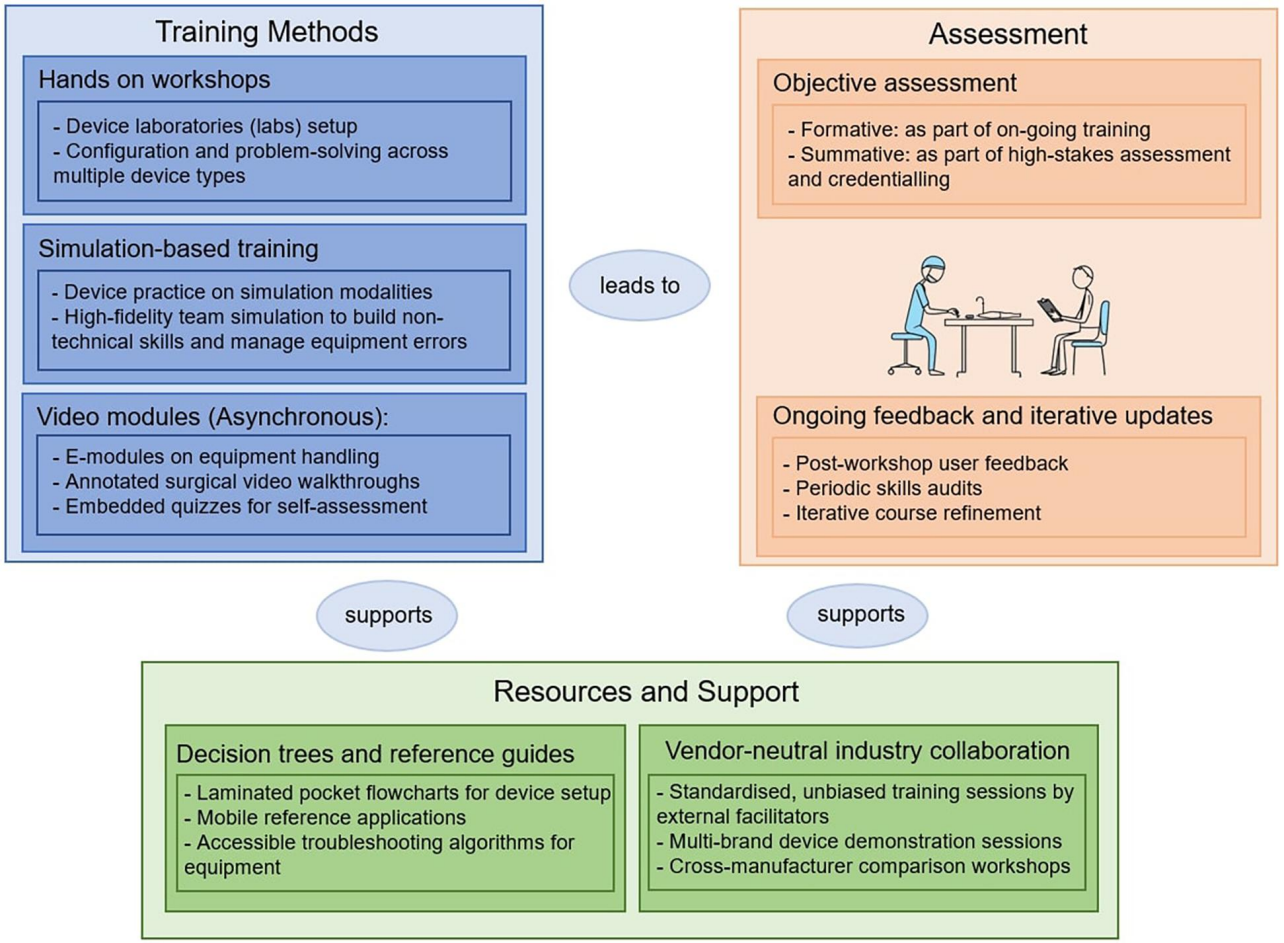
Practical elements and recommendations included in the TEC toolkit.

Figure 2 presents the proposed ECT Toolkit, developed in direct response to the educational gaps and priorities identified through this study. Component selection was grounded in quantitative survey data and inductive thematic analysis, rather than chosen arbitrarily (Supplementary Files S2, S3). High or consensus agreement on the value of SBT in this context, multimedia modules, decision aids, and hands-on workshops, combined with strong support for industry engagement, multidisciplinary training, and structured assessment, collectively shaped the Toolkit's design. This approach ensured the model reflected both expert opinion and the practical realities of TEC-driven training.

As next steps, phased implementation of the TEC Toolkit should begin with targeted pilot workshops for high-risk TEC components, accompanied by structured evaluation using defined outcome measures. These include competency benchmarks mapped to curriculum milestones, patient safety indicators derived from simulation debrief outcomes, and user satisfaction metrics capturing learner and faculty perspectives. Pilot programmes can be delivered through regional simulation centres, integrating pre- and post-training assessments of confidence, skill proficiency, and transfer to clinical practice. Continuous refinement will follow an iterative “plan–implement–evaluate–refine” model, drawing on established implementation science frameworks used in other validated surgical education initiatives such as the OPTI-Surg project (25). Longitudinal monitoring of trainee performance and educational impact will support continuous improvement and sustainable integration across institutions.

## Strengths and limitations

This study represents the first national, cross-specialty survey in Ireland to explore surgical educator perspectives on TEC training. Participants included senior educators with formal roles in curriculum design and simulation leadership, ensuring that the findings reflect expert, system-level insights. The study also achieved broad specialty coverage, including 12 surgical disciplines that frequently operate in technologically complex environments. The combination of quantitative and qualitative data enabled both consensus measurement and thematic exploration, enhancing the robustness and contextual relevance of the findings. Importantly, the development of the TEC Toolkit was directly informed by these data, reinforcing its practical applicability and educational alignment.

However, the study also has limitations. It relied solely on educator perspectives and did not include input from trainees or patients. As a self-report survey, the findings are subject to recall bias, whereby participants' retrospective accounts may be shaped by selective memory or interpretation. Responses were also based on perceptions rather than observed behaviours, which may introduce reporting bias. The relatively small number of respondents per specialty, while nationally representative, may limit specialty-specific generalisability. The use of Likert-scale items, although appropriate for consensus analysis, may not fully capture the complexity of TEC training challenges. Additionally, the cross-sectional design limits insight into how attitudes or practices evolve over time. Broader stakeholder engagement, observational studies, and longitudinal evaluation will be essential for assessing the impact and scalability of the proposed Toolkit.

## Conclusion

This study highlights an often-overlooked dimension of surgical education: the holistic readiness required for safe and confident engagement with TEC in the OT. The national survey demonstrated clear, cross-specialty consensus on the urgent need for structured TEC training. The proposed TEC Toolkit, grounded in educational theory and expert insight, provides a practical and scalable solution to this gap. Broadening the concept of surgical readiness to include TEC literacy, contextual equipment use, and SBT exposure will better prepare future surgeons for the technological demands of modern practice. Although developed within a national context, the challenges identified are globally relevant, and the TEC Toolkit offers a flexible model that can be adapted to diverse international training environments, including those in low resource settings.

## Data Availability

The original contributions presented in the study are included in the article/Supplementary Material, further inquiries can be directed to the corresponding author.

## Supplementary material

Supplementary File 1 Codebook used in thematic analysis of free-text survey responses, including code definitions, example excerpts, and theme classifications to support transparency and reproducibility of qualitative findings.

Supplementary File 2 Median, Frequency, and Percentage Distributions of Responses from Questionnaire Items.

Supplementary File 3 Median (IQR) scores and Kruskal–Wallis p-values for TEC survey items by surgical specialty group
