# Supplementary material for: Preparing surgeons for the modern operating theatre: insights from a national survey on technology use and readiness
Source: Front Surg. 2025 Nov 14;12:1686653. doi: 10.3389/fsurg.2025.1686653 (PMC12661567; doi:10.3389/fsurg.2025.1686653)
Supplement: Supplementary File 2 — Median, Frequency, and Percentage Distributions of Responses from Questionnaire Items. [file Supplementaryfile2.docx]

| **Framework Link (Kern + Constructivist)** | **Questionnaire Instrument** | **Strongly Disagree *n(*%)** | **Disagree *n*(%)** | **Neither Agree Nor Disagree *n*(%)** | **Agree *n*(%)** | **Strongly Agree *n*(%)** | **Median (IQR)** |
| --- | --- | --- | --- | --- | --- | --- | --- |
|  | A. Perspectives |  |  |  |  |  |  |
| Kern Step 1: Identifies the overarching problem and its link to patient safety; Constructivist: builds on practitioners’ shared understanding from experience | Technology, equipment, consumables (TEC) readiness is essential for ensuring patient safety during surgery | 7 (20%) | 0 (0%) | 1 (3%) | 8 (23%) | 19 (54%) | 5 (4, 5) |
| Kern Step 2: Targets learner needs by drawing on real-world incidents; Constructivist: uses reflective practitioner experience to highlight learning gaps | Lack of TEC familiarity among trainees has contributed to *near-misses or *adverse events in the OT in my experience | 3 (9%) | 4 (11%) | 9 (26%) | 15 (43%) | 4 (11%) | 4 (3, 4) |
| Kern Step 3: Assesses curriculum alignment with desired competencies; Constructivist: encourages evaluation of formal instruction against practice realities | The current ISCP curriculum (or equivalent) adequately covers TEC-related competencies relevant to patient safety | 0 (0%) | 16 (46%) | 12 (34%) | 7 (20%) | 0 (0%) | 3 (2, 3) |
| Kern Step 4: Explores educational strategies in use; Constructivist: values learning through repeated, contextualised simulation practice | Trainees receive regular simulation-based exposure to the surgical devices and technologies most commonly used in our OT | 0 (0%) | 20 (57%) | 7 (20%) | 7 (20%) | 1 (3%) | 2 (2, 3) |
| Kern Step 6: Evaluates the perceived effectiveness of training; Constructivist: emphasises feedback from applied practice to shape learning | Simulation-based TEC training has a measurable impact on reducing trainee errors or hesitations during live surgery | 0 (0%) | 0 (0%) | 7 (20%) | 24 (69%) | 4 (11%) | 4 (4, 4) |
|  | B. Current practices |  |  |  |  |  |  |
| Kern Step 5: Examines organisational capacity for implementation; Constructivist: recognises the importance of supportive learning environments | There is sufficient institutional support for incorporating TEC-focused training with an emphasis on operative competence and patient safety | 0 (0%) | 13 (37%) | 11 (31%) | 9 (26%) | 2 (6%) | 3 (2, 4) |
| Kern Step 4: Investigates specific teaching strategies; Constructivist: focuses on situated learning under realistic conditions | Our programme uses effective methods to teach TEC usage, troubleshooting, and decision-making under real-life OT pressures | 1 (3%) | 11 (31%) | 13 (37%) | 10 (29%) | 0 (0%) | 3 (2, 4) |
| Kern Step 5: Identifies practical barriers to implementation; Constructivist: acknowledges context as a key factor in learning feasibility | Time constraints in hospital practice / the curriculum, limit our ability to include comprehensive TEC training focused on patient safety | 0 (0%) | 4 (11%) | 0 (0%) | 23 (65%) | 8 (23%) | 4 (4, 5) |
| Kern Step 2: Needs assessment for resource adequacy; Constructivist: addresses authentic access to tools as central to skill acquisition | Limited access to up-to-date surgical equipment hampers our ability to teach TEC skills relevant to current OT practice | 1 (3%) | 8 (23%) | 3 (9%) | 18 (51%) | 5 (14%) | 4 (3, 4) |
| Kern Step 6: Highlights gaps in evaluation and feedback processes; Constructivist: values post-event reflection as part of deep learning | There is a lack of structured incident reporting or debriefing related to TEC issues in our training programme | 0 (0%) | 4 (11%) | 3 (9%) | 23 (66%) | 5 (14%) | 4 (4, 4) |
|  | C. Needs |  |  |  |  |  |  |
| Kern Step 3 & 4: Sets specific learning objectives and strategies; Constructivist: supports collaborative, shared learning resources | Our programme would benefit from a nationally developed TEC toolkit with a focus on developing operator competence and patient safety | 0 (0%) | 1 (3%) | 0 (0%) | 25 (71%) | 9 (26%) | 4 (4, 5) |
| Kern Step 3: Defines agreed learning goals; Constructivist: aligns shared standards with practitioner consensus | I would support the development of national guidelines on TEC education in surgical training | 0 (0%) | 0 (0%) | 1 (3%) | 24 (69%) | 10 (28%) | 4 (4, 4) |
| Kern Step 3: Establishes intended outcomes; Constructivist: integrates structured, scaffolded learning for readiness | A structured TEC curriculum would improve overall trainee readiness and potentially reduce OT errors | 0 (0%) | 0 (0%) | 5 (14%) | 21 (60%) | 9 (26%) | 4 (4, 4) |
| Kern Step 4: Specifies preferred educational strategies; Constructivist: promotes active, experiential learning | Hands-on workshops for learning and troubleshooting TEC should be a standard part of surgical training | 0 (0%) | 0 (0%) | 2 (6%) | 18 (51%) | 15 (43%) | 4 (4, 5) |
| Kern Step 4: Suggests blended learning methods; Constructivist: supports flexible, learner-controlled engagement | Video-based modules on device set-up, usage, and common pitfalls are important for improving patient safety in surgical training | 0 (0%) | 2 (6%) | 1 (3%) | 21 (60%) | 11 (31%) | 4 (4, 5) |
| Kern Step 4: Endorses job aids as part of strategy; Constructivist: facilitates just-in-time learning in context | Quick-reference guides or decision trees for TEC selection and troubleshooting should be available to all surgical trainees | 0 (0%) | 3 (9%) | 3 (9%) | 21 (60%) | 8 (23%) | 4 (4, 4) |
| Kern Step 4: Advocates interprofessional simulation; Constructivist: uses authentic team contexts to reinforce skills | Multidisciplinary simulation sessions (including OT nurses and technicians) are valuable for practicing team-based responses to TEC-related incidents | 0 (0%) | 0 (0%) | 3 (9%) | 14 (40%) | 18 (51%) | 5 (4, 5) |
| Kern Step 5: Leverages external partnerships for implementation; Constructivist: incorporates diverse expertise into situated learning | Collaboration with industry for device demonstrations and training on new safety features should be integrated into surgical education | 0 (0%) | 0 (0%) | 3 (9%) | 14 (40%) | 18 (51%) | 4 (4, 5) |
| **Near-miss: an incident that could have caused harm to a patient but did not, either by chance or because it was caught in time* | | | | | | | |
| **Adverse event: an incident where harm actually occurred to the patient as a result of medical care, not the underlying condition* | | | | | | | |
