# Supplementary material for: Preparing surgeons for the modern operating theatre: insights from a national survey on technology use and readiness
Source: Front Surg. 2025 Nov 14;12:1686653. doi: 10.3389/fsurg.2025.1686653 (PMC12661567; doi:10.3389/fsurg.2025.1686653)
Supplement: Supplementary File 3 — Median (IQR) scores and Kruskal–Wallis p-values for TEC survey items by surgical specialty group [file Supplementaryfile3.docx]

|  |  |  |  |  |
| --- | --- | --- | --- | --- |
| **Questionnaire instrument** | **Group 1: Median (IQR)** | **Group 2: Median (IQR)** | **Group 3: Median (IQR)** | **P-value** |
| A. Perspectives |  |  |  |  |
| The current ISCP curriculum (or equivalent) adequately covers TEC-related competencies relevant to patient safety | 3 (2, 4) | 2 (2, 3) | 3 (2, 3) | 0.77 |
| Trainees receive regular simulation-based exposure to the surgical devices and technologies most commonly used in our OT | 4 (2, 4) | 2 (2, 3) | 2 (2, 3) | 0.15 |
| B. Current practices |  |  |  |  |
| There is sufficient institutional support for incorporating TEC-focused training with an emphasis on operative competence and patient safety | 3 (2, 4) | 3 (2, 4) | 3 (2, 4) | 0.89 |
| Limited access to up-to-date surgical equipment hampers our ability to teach TEC skills relevant to current OT practice | 4 (2, 4) | 4 (2, 4) | 4 (4, 5) | 0.46 |
| There is a lack of structured incident reporting or debriefing related to TEC issues in our training programme | 4 (3, 4) | 4 (4, 4) | 4 (4, 5) | 0.05 |
| C. Needs |  |  |  |  |
| Multidisciplinary simulation sessions (including OT nurses and technicians) are valuable for practicing team-based responses to TEC-related incidents | 4 (4, 5) | 5 (4, 5) | 5 (4, 5) | 0.18 |
| Collaboration with industry for device demonstrations and training on new safety features should be integrated into surgical education | 5 (4, 5) | 4 (4, 5) | 4 (4, 5) | 0.21 |
|  | | |  | |
